# Four Classes of Dentists

**Published:** 1898-04

**Authors:** 


ARTICLE II.

### FOUR CLASSES OF DENTISTS.

By analysis it may be discovered that instead of two classes of dentists, as commonly supposed, Professional men and Quacks, there are already four, viz.: The true professional man; the Quack Professional; the Professional Quack, and the Quack.

The true professional man, in the highest and noblest sense of the words, is a *rara avis*. He begins practice just as the true minister of the gospel takes up preaching. He is prompted by a love of his work, and a love for his fellow-man. That he earns a living by the fees which he obtains, in no sense detracts from his attitude of being a missionary. He must live, that he may continue his mission. At times he receives high fees. Indeed such men receive the very highest fees, for the people are not all fools, and merit will eventually be recognized, and its reward cheerfully given. But the professional man, while exacting large fees from the rich, takes smaller ones from the less wealthy, and perhaps nothing at all from the needy. But even in his treatment of the very rich, he acts in a professional manner. He has in his own mind a fixed valuation for the service rendered. If the patron can afford this maximum sum, it is demanded. But if the next applicant for a similar service should own ten millions instead of one, the fee would be no greater. When a patient is entrusted to his care, the thought uppermost is not, "How much can I make out of this patient," but, rather, "How much can I accomplish; how much comfort can I afford to this sufferer?" In all services rendered the highest standard is ever before him. Whatever method promises the most lasting benefit he pursues without regard to time, money, or personal convenience. Many men fill teeth with amalgam, and preach the great advantage of using this material in preference to gold, who adopt such a course from laziness, and they write their articles to quiet a troublesome conscience, like the coward in the dark, who held one of his hands with the other trying to believe that he was not alone. Such a man is not truly professional.

The true professional man, then, is that man who is especially adapted to all the demands of his calling, both moral and mental. He has ever prominent in his mind the best interests of his patient, of his fellow practitioners, and of his profession which, by every act, he endeavors to lift to a higher plane in the esteem of the community. He conducts himself at all time so that his patrons place themselves, their wives and daughters, in his care with confidence in his integrity, and faith in his skill.

In New York City there resides one dentist who represents the highest type of the professional man.

Among those who claim to be professional men will be found men who are professional in varying degrees, until at the opposite extreme we find the quack professional. The quack professional, is like the crow who daubed himself with white paint and flocked with the geese, that the farmer might not suspect that he had one eye on the growing corn. This man is in dentistry for what there is in it. He poses with the professional men because perhaps by education and social position he naturally desires a clientele among the higher classes. He has no wish to work for the poor. It is the purse of the millionaire which is his constant aim. Such a man recently told a young man: "You cannot afford to have your teeth put in order." In desperation the young man sought advice elsewhere and cheerfully paid two hundred dollars to the gentleman who saved his teeth. Another boasted in a dental society meeting that he was "Not in dentistry for the good of his health," and the applause which greeted the remark proved that there were many present who had no very high estimate of professional rectitude.

While posing as a true professional man, the quack professional is his direct opposite in the matter of advertising. He does not advertise in the public prints. He is too shrewd for that. He knows the Code, and is very careful never to overstep the bounds. But one may advertise in many ways, not specially interdicted by the Code of Ethics. This man, unlike the true professional, is always *bragging*. He never losses an opportunity to explain how much greater he is than his fellows; how much more skillful; how many discoveries he has made; how many methods devised; how many instruments invented; and in short how very much indebted to him is all the rest of the dental world.

He also understands how to use printer's ink. He gets printed advertisements without paying for them. The chief object of advertising is to increase business. Consequently any use of the printer's art which accomplishes this end is advertising, however cleverly concealed. Indeed in these days, when advertising has grown to be a special study, the concealment of the advertisement is supposed to be the highest art. So our quack professional; our crow flocking with the geese who do not observe the black feathers beneath the white coat of paint, usually pursues some specialty, or pretends to do so; generally he practices everything, "from fillings to false teeth," but announces that he is especially successful in one line or practice. He writes many learned theses, some of which may be actually valuable additions to science, which fact adds to rather than detracts from their value as advertisements. His paper is usually read before a dental society, at which the author has a fine opportunity to pose as the special disciple of the methods advocated; next it appears in the most widely read journal. Would it not seem that this is efficient? It is not, as viewed from the standpoint of the quack professional. He is not yet sure that it has reached those places where it will do the most good; therefore he orders one or two thousand reprints, and these are industriously distributed over that field from which he garners his corn. Of course it is not intended here to state that all men who distribute reprints, are of this class. Many do this, as they do other work, without thought of self. But there is a little sign by which the pamphlet of the quack professional can always be detected. At the end of the last page (if not on the first page of the cover) appears his office address. He is not willing that the reader should have any difficulty in finding him. When next you receive a reprint, bearing this little ear mark, think of the pamphlet as white paint and look for the crow.

The professional quack is a miserable sort of fellow. He takes a notion tha he would like to be a dentist. He has little or no education, but in these days that is no obstacle, providing he has a couple of hundred of dollars. Any dental college in the United States will accept him as a student (*vide* proceedings National Association of Dental Faculties). He worries along through college to the examination period, and this he passes, because he wears long white cuffs, and knows how to write a fine legible hand with a well pointed pencil. So he gets through and is awarded a diploma. He does not send that pair of cuffs to the laundry, but uses them at a State Board examination which he passes with the same facility, and with the same lack of knowledge as when going through college. Thus he obtains his license. The college and the State Board have done all that they could for him; they have launched him. But thrown upon his own resources he finds that he has none. So one day his room rent is due, and his lunch ticket is punched full of holes, and if he ever heard any lecture on the subject of upholding the dignity of his profession, or if he ever made any promise to do so, he forgets it all. He hires himself to a quack and becomes one of the "professors" in a "Cash Dental Parlor." Poor devil, is it his fault? Of course he ought to have taken a position in a carpenter shop, if he had any mechanical skill or perhaps in a planing mill or machine shop. But his little learning toward mechanics made him imagine himself fitted for dentistry, and when he applied for admission into college *the professors did not undeceive him.* Perhaps a college which only accepted high grade, well educated, refined men as students, would not declare large dividends to its stockholders, but what a work its graduates would do in the world!

Of professional quacks (who, mind you, are only quacks until they get a start), there are hundreds in New York, and thousands about the country.

The simon pure quack, when you come to know him, may not be half a bad fellow. Of course he is not a professional man; but then he does not pretend that he is. All he asks is permission to do business in his own way. And strangely enough, the very laws which have been passed to elevate the profession (what a pleasant phrase that is as it rolls off the tongue) and presumably to suppress quackery, have had exactly the reverse effect. The quack is freer to go and come to day, than your most cultured professional man. Suppose we prove that? At the present moment an old practitioner is intending to leave the north because his health demands a warmer climate. He probably will settle in Texas, *if he can remember enough of what he learned at college to pass the Texas Board.* He has a chance to dispose of his practice to a widely-known dentist in New England. *It all depends upon the success of the latter with the New York State Board.* Thus the real professional men are hampered, or else confined to practice in one State. How with Mr. Quack? In January he opens a place of business in New York. In February he starts a branch in Philadelphia. In March he opens another in Boston, and in May he begins business in Chicago. He passes *none* of the State Board examinations. Why? *Because he is not practicing dentistry, he is only doing business.* His staff of operators are the professional quacks, the poor devils whom the colleges have turned out as dentists, and who have been licensed by the State Boards. He can find dozens of such men in every large city. Consequently his sphere of action is limited only by his capacity for managing a concern with many branches. In short, the quack is merely a man of business, taking advantage of the opportunity which the wise men in the dental ranks have afforded them by enacting laws, which enable them to run shops, in which all the operators have been declared legally qualified. He may not be a dentist at all.

Of this class there are hundreds—and more start in business every year.—Dr. Ottolengui, *Editorial Items of Interest.*

